# PD11 - Omalizumab in children: experience in the Immunoallergology Department

**DOI:** 10.1186/2045-7022-4-S1-P11

**Published:** 2014-02-28

**Authors:** Rita Aguiar, Pedro Silva, Fátima Cabral Duarte, Ana Mendes, Ana Célia Costa, Manuel Barbosa Pereira

**Affiliations:** 1 Department of Immunoallergology, Hospital de Santa Maria, CHLN, Lisbon, Portugal

## Introduction

Omalizumab is a monoclonal antibody approved for the treatment of severe allergic asthma for patineis above 6 years old in Europe, but its use has been described successfully in other IgE-mediated diseases.

## Objective

Evaluation of clinical response and safety of treatment with omalizumab in a pediatric population.

## Methods

Retrospective analysis of clinical data of pts aged <18 years at the time of initiation of treatment with omalizumab in our department from December 2009 to July 2013.We evaluate adverse reactions, clinical outcome, reduction or discontinuation of therapy according to allergic disease.

## Results

Ten pts were proposed to omalizumab: 8M, 2F with a mean age of 12.9 years ±3.9 [7-17]. All had bronchial asthma: 3severe allergic asthma, 5severe atopic eczema (AE), 1severe ocular allergy (OA) with risk for injury to the cornea by conjunctival edema and 1 cow's milk protein allergy (CMA) with anaphylaxis during the protocol for oral tolerance induction (OTI).

The mean duration of therapy with omalizumab was 12.3±13.35 months. The median serum total IgE l before treatment was 991.5kU/L.

The average values of Asthma Control Test (ACT) before and after omalizumab were 17 [15-24]. The mean Scoring Atopic Dermatitis (SCORAD) before and after omalizumab was 2 [34-90] and 24 [13-35].

The pts with EA, 1 was treated with cyclosporine, 1 azathioprine, 2 with systemic corticosteroids (SC). After an average of 2 months of treatment there was a reduction of azathioprine and after an average of 5.3 months suspension of SC and cyclosporine.

Two pts that had suspended Specific immunotherapy (SI) for clinical worsening before omalizumab re-started SI without occurrence of adverse reactions.

There were no adverse reactions to omalizumab.

## Discussion

In our pediatric population, omalizumab shown to be effective and safe in patients with severe uncontrolled allergic disease, not only on asthma but also in other pathologies, allowing application of other effective therapeutics such as induction of food intolerance and SI that can change the course of the disease. Although more studies are still needed but this antibody appears to have a promising role.

